# Animal Chemistry; or Organic Chemistry in Its Application to Physiology and Pathology

**Published:** 1842-10-01

**Authors:** 


### Medico-Chirurgical Review,

### No. LXXIV.

**[No. 34 of a Decennial Series.]**

JULY 1, to OCTOBER 1, 1842.

Animal Chemistry; or Organic Chemistry in its applications to Physiology and Pathology. By *Justus Liebig*, M.D. &c. Edited by *William Gregory*, M.D. London, Taylor and Walton, 1842.

This truly interesting work consists of three parts. The first part contains a minute examination of the processes employed in the nutrition and re-production of the various structures of the animal economy. In animals and vegetables we recognize the existence of a certain force, the source of growth and of re-production, in a state of rest, or, to use the author's term, of static equilibrium. This state of rest becomes disturbed by the action of external influences, by impregnation, and the presence of air and moisture; by these influences, it enters into a state of motion or activity, and exhibits itself in the production of forms. To this force the appellation of *vital force,* or *vitality,* has been given. The growth of a vegetable is effected by a decomposition which takes place in some parts of it under the influence of light and heat; and it is to be observed that it is exclusively inorganic matter, which is thus decomposed; and if with several eminent mineralogists we consider atmospheric air to be a mineral, we may lay it down that the vital process in vegetables effects the transformation of mineral substances into a living organism. The increase of mass in a living plant implies that certain component parts of its nourishment become component parts of the plant; the fact is now established, that the growth of plants depends on the elimination of oxygen, which is separated from the other component parts of the nourishment, whilst the life of animals exhibits itself in the continual absorption of oxygen, and its combination with certain component parts of the animal body. Another distinction between animal and vegetable life is that, whilst vegetables require inorganic matter as food, animals, on the contrary, require highly organized atoms for their support. They can, in fact, only subsist upon parts of an organism. Animals are distinguished from vegetables by the faculty of locomotion, and, in general, by the possession of senses. These distinguishing faculties depend on the presence of a nervous apparatus, of which vegetables are destitute. The phenomena of motion in vegetables, as the circulation of the sap and the closing of flowers and leaves, depend on mechanical causes. Assimilation, or the process of growth, goes on in the same way in animals and vegetables. In both, the same cause determines the increase of mass. This constitutes the true vegetative life, which is carried on without consciousness. Pathology informs us that the true vegetative life in animals is not dependent on the presence of this nervous apparatus; as the process of nutrition proceeds in those parts of the body where the nerves of sensation and voluntary motion are paralysed; whilst, on the other hand, the most energetic volition is unable to exert any influence on the contractions of the heart, on the motion of the intestines, or on the processes of secretion.

The phenomena of mind, of the proximate or ultimate causes of which we know nothing, we ascribe to an immaterial agency, one which, so far as its manifestations are connected with matter, is entirely distinct from the vital force, with which it has nothing in common. This force, however, exerts a certain influence on the activity of vegetative life, an influence by no means of a determinative kind, as it merely accelerates, retards, or disturbs the process of vegetative life. In a manner exactly analogous, the vegetative life re-acts on the conscious mental existence.

All the parts of the animal body are produced from a peculiar fluid circulating in its organism, by virtue of the vital force residing in every organ or part of an organ. All the parts of the body were originally blood, or at least these parts were brought to the growing organs by means of the blood. At each moment of life a continued change of matter is going on in the animal organism; a part of the structure is transformed into unorganised matter, loses its condition of life, and must be again renewed. Every motion, every manifestation of force, is the result of a transformation of the structure, or of its substance. Every conception, every mental affection is followed by changes in the chemical nature of the secreted fluids; every thought, every sensation, is accompanied by a change in the composition of the substance of the brain. To keep up the phenomena of life in animals, nourishment is required; this serves either for the increase of the mass, (nutrition,) or for the supply of the matter consumed, (re-production,) or for the production of force.

The first condition of animal life is the assimilation of food, and the second is the continual absorption of oxygen from the atmosphere.

All vital activity results from the mutual action of the oxygen of the atmophere and the elements of the food. All changes proceeding in the body are decidedly of a chemical nature, although they are sometimes increased or diminished in intensity by the influence of the vital force. The action of poisons and of medicinal agents on the living animal body shews that the chemical decompositions and combinations which manifest themselves in the phenomena of vitality, may be influenced by bodies having a well-defined chemical action. As in the closed galvanic circuit, in consequence of certain changes which an inorganic body, a metal, undergoes when placed in contact with an acid, a certain something becomes cognizable by our senses, which we call a current of electricity; so in the animal body, in consequence of transformations undergone by matter previously constituting a part of the organism, certain phenomena of motion and activity are perceived; and these are called life or vitality. The electrical current manifests itself in certain phenomena of attraction and repulsion, which it excites in other bodies naturally motionless, and by the phenomena of the formation and decomposition of chemical compounds, which occur every where, when the resistance is not sufficient to arrest the current. It is from this point of view and no other that chemistry ought to view the various phenomena of life. The first conditions of animal life are nourishment and oxygen introduced into the system.

According to Lavoisier, an adult man takes into his system from the atmosphere, in one year, from 700 to 800lbs. of oxygen; and yet he does not increase in weight. What becomes of the enormous weight of oxygen thus introduced in the course of a year into the human system? The answer is: the carbon and hydrogen of certain parts of the body have entered into combination with the oxygen introduced through the lungs and through the skin, and have been given out in the forms of carbonic acid gas and the vapour of water. At every moment, with every expiration, certain parts of its elements are separated from the animal organism, after having entered, within the body, into combination with the oxygen of the atmosphere. It is ascertained that an adult inspires $32\frac{1}{2}$ ounces of oxygen daily, $=$ 15,661 grains, French weight, and that the weight of the whole mass of his blood, of which 80 per cent is water, is 24lbs.; accordingly there are in the body of the adult 4·8lbs. of dry blood, for the combustion of the carbon and hydrogen of which 64,103 grains of oxygen are required. Thus, then, pre-supposing the weight of the body to remain unchanged, the body of a man who takes daily into the system $32\frac{1}{2}$ ounces of oxygen, must receive daily in the shape of nourishment as much carbon and hydrogen as would suffice to supply 24lbs. of blood with these elements. This supply is derived from the food. It appears that an adult taking moderate exercise consumes as much food as yields about 14 ounces of carbon daily, which require 37 ounces of oxygen for their conversion into carbonic acid.

Since no part of the oxygen inspired is again expired as such, but is given off as a compound of carbon or hydrogen, and also since the carbon and hydrogen given off must be replaced by carbon and hydrogen derived from the food, it follows that the amount of nourishment required in the animal body is directly proportional to the quantity of oxygen taken into the system; or what comes to the same thing, as the quantity of oxygen taken in may be expressed by the number of respirations, the quantity of nourishment required must vary with the number and force of the respirations; thus a child in whom the respiratory organs are naturally very active, requires food oftener than an adult, and bears hunger less patiently. A bird deprived of food dies on the third day, while a serpent, with its sluggish respiration, can live without food three months and more. For the same reason, the quantity of food required by an individual in a state of rest is less than that required during exercise or work, the number of respirations being smaller in the former case than in the latter. The quantity of oxygen inspired is also affected by the temperature and density of the atmosphere. The capacity of the chest in an animal being a constant quantity, it is evident that he takes in the same *volume* of air at each inspiration; hence as air is expanded by heat and contracted by cold, equal volumes of hot and cold air contain unequal weights of oxygen; in Summer also, the air contains aqueous vapour, whilst in Winter it is dry, that is, it contains, for the same volume, more oxygen in Winter than in Summer. For the same reason, in an equal number of respirations we consume more oxygen at the level of the sea than on a mountain; hence the quantity of oxygen inspired and of carbonic acid expired must vary with the height of the barometer. The oxygen taken into the system being given out in the same forms, whether it be Summer or Winter, we expire more carbon in cold weather than we do in warm weather; and we must consume more or less carbon in our food in the same proportion; in our climate an eighth more in Winter than in Summer. Even when equal weights of food are consumed in warm and cold countries, infinite wisdom has so arranged it that the articles of food in different climates yield very unequal proportions of carbon. The fruits on which the natives of the South prefer to feed do not contain more than 12 per cent. of carbon, while the bacon and train oil used by the inhabitants of the Arctic regions contain from 66 to 80 per cent. of carbon.

The mutual action between the elements of the food and the oxygen conveyed by the circulation of the blood to every part of the body is the *source of animal heat.* All living creatures, whose existence depends on the aborption of oxygen, possess within themselves a source of heat independent of the medium in which they live. This heat is the result of the combination of a combustible substance (carbon and hydrogen) with oxygen—we see that animal heat is produced only in those parts of the body to which arterial blood, and with it the oxygen absorbed in respiration is conveyed. The carbon and hydrogen of the food, in being converted by oxygen into carbonic acid and water, must give out as much heat as if they were burned in the open air. The only difference is, that the amount of heat produced is diffused over unequal spaces of time. It is obvious that the amount of heat liberated must increase or diminish with the quantity of oxygen introduced in equal times by respiration. Those animals which respire frequently, and consequently consume much oxygen, possess a higher temperature than others, which, with a body of equal size to be heated, take into the system less oxygen. The temperature of the human body is the same in the torrid as in the frigid zone. But as the body may be considered in the light of a heated mass, which cools with an accelerated rapidity the colder the surrounding medium is, it is evident that the fuel necessary to sustain its heat must vary in different climates. Hence we see how unequal the loss of heat must be in a man at Palermo, where the external temperature is nearly equal to that of the body, and in the polar regions, where the external temperature is from 70° to 90° lower. The supply of heat lost by cooling is effected by the mutual action of the elements of the food and the inspired oxygen, which combine together. The animal body acts, in this respect, as a furnace, which we supply with fuel. In order to keep up in the furnace a constant temperature we must vary the supply of fuel according to the external temperature, that is, according to the supply of oxygen. Now in the animal body the food is the fuel; with a proper supply of oxygen we obtain the heat given out during its combustion. In Winter, when we take exercise in a cold atmosphere, and when therefore the amount of inspired oxygen increases, the necessity for food containing carbon and hydrogen increases in the same ratio, and by gratifying the appetite thus excited, we obtain the most efficient protection against the cold. A starving man is soon frozen to death; and it is well known that the animals of prey in the arctic regions far exceed in voracity those of the torrid zone.

Our clothing is an equivalent for a certain amount of food. The more warmly we are clad, the less urgent becomes our appetite for food, the loss of heat by cooling, and consequently the amount to be supplied by the food, being diminished. If we went naked, like some savage tribes, or if in hunting or fishing we were exposed to the same degree of cold as the Samoyedes, we should be able with ease to consume 10lbs. of flesh, and perhaps a dozen of tallow-candles into the bargain, daily, as warmly-clad travellers have related with astonishment of these people. We should then be able to take the same quantity of brandy or train oil without bad effects, because the carbon and hydrogen of these substances would only suffice to keep up the equilibrium between the external temperature and that of our bodies. Thus then we may lay it down, that the quantity of food is regulated by the number of respirations, by the temperature of the air, and by the amount of heat given off to surrounding objects. We now have an explanation of the apparently anomalous habits of different nations. The colder the region, the more combustible must the food be. The Italian cannot, with impunity, take more carbon and hydrogen in the shape of food than he expires as carbonic acid and water; nor can the Greenlander expire more carbon and hydrogen than he takes into the system as food, unless in a state of disease or starvation. The Englishman in Jamaica perceives with regret the disappearance of his appetite, previously a source of frequently recurring enjoyment; and he succeeds by the use of aromatics and powerful stimulants in enabling himself to take as much food as he was accustomed to at home. But he thus unfits himself for the climate in which he is placed; for sufficient oxygen does not enter the system to combine with the carbon introduced into the system; some of this carbon therefore remains unconsumed; and the heat of the climate prevents him from taking exercise so as to increase the number of his respirations, and thus to proportion the waste to the amount of food taken; the consequence of this then is disease of some kind, the unconsumed carbon being forced into other channels. On the other hand, England sends her dyspeptic invalids to southern regions. In their native climate their impaired digestive organs are unable to fit the food for that state in which it best unites with the oxygen of the air, which therefore acts on the respiratory organs themselves. When they are removed to a warmer climate, they absorb less oxygen and take less food; and the diseased organs of digestion have sufficient power to place the diminished amount of food in equilibrium with the respired oxygen. In conformity with these views in our own climate, hepatic diseases, or diseases arising from excess of carbon, are more prevalent in Summer, and in Winter pulmonic diseases, or those arising from an excess of oxygen.

We have here assumed that the production of animal heat is effected more especially by the combination of carbon and hydrogen with oxygen. The entire process of respiration appears most clearly developed when we consider the state of a man or other animal totally deprived of food. The first effect of starvation is the disappearance of fat, and this fact cannot be traced either in the urine or fæces. Its carbon and hydrogen have been given off through the skin and lungs in the form of oxidized products; they must have served to support respiration. In the case of a starving man $32\frac{1}{2}$ ounces of oxygen enter the system daily, and are given out again in combination with part of his body. The whole history of hybernating animals, and the well-established facts of the periodical accumulation, in various animals, of fat, which, at other periods, entirely disappears, prove that the oxygen, in the respiratory process, consumes, without exception, all such substances as are capable of combining with it. It combines with whatever is presented to it; and the deficiency of hydrogen is the only reason why carbonic acid is the chief product.

In the progress of starvation, it is not only the fat which disappears, but also, by degrees, all such of the solids as are capable of being dissolved; such as the muscles which become shrunk and soft. Towards the end the particles of the brain begin to undergo the process of oxidation, and delirium, mania and death close the scene; that is, all resistance to the oxidizing power of the atmospheric oxygen ceases, and the chemical process of decay commences, in which every part of the body, except the bones, enters into combination with oxygen. The time required to cause death by starvation depends on the amount of fat in the body, on the degree of exercise, on the temperature of the air, and, lastly, on the presence or absence of water. In some cases, where a full supply of water was accessible to the sufferer, death has not occurred till after a lapse of twenty days.

In all chronic diseases death is produced by the same cause; viz. by the chemical action of the atmosphere. In the absence of those substances, whose function in the organism is to support the process of respiration; when the diseased organs are incapable of performing their proper function of producing these substances; when they have lost the power of transforming the food into that shape in which it may, by entering into combination with the oxygen of the air, protect the system from its influence, then the substance of the organs themselves, the fat of the body, the substance of the muscles, the nerves and the brain, are unavoidably consumed. The action of the air is the true cause of death in these cases.

A deficiency of food and a want of power to convert the food into a part of the organism are both, equally, a want of resistance; and this is the negative cause of the cessation of life. The flame is extinguished because the oil is consumed, and it is the oxygen of the air which has consumed it.

Some have attributed a part of the heat generated in the animal body to the action of the nervous system. Professor Liebig says that if this view exclude chemical action, or changes in the arrangement of the elementary particles, as a condition of nervous agency, it means nothing else than to derive the presence of motion, the manifestation of a force, from nothing. The idea that the production of animal heat is purely the result of nervous agency seems to have arisen from the notion that the inspired oxygen combines with the carbon in the blood itself. Nothing can however be more erroneous than such an idea. That the nervous system has a share in the respiratory function no one will deny; as no change of condition can occur in the body without the nerves, they being essential to all motions. It is under their influence the viscera produce those compounds, which, while they protect the organism from the action of oxygen, give rise to animal heat. On cutting through the pons Varolii, the spinal cord, or par vagum, the respiratory motions continue for some time; but the oxygen does not meet with those substances with which, in the normal state, it would have combined: because the paralyzed viscera no longer furnish them. The author next proceeds to prove that animal heat is not indebted for its production to muscular contraction. The contraction of muscles produces heat; but the force necessary for such contraction has manifested itself through the organs of motion, in which it has been excited by chemical changes. The ultimate cause of the heat produced is therefore to be found in these chemical changes.

There are various causes by which force or motion may be produced. A bent spring, a current of air, the fall of water, fire applied to a boiler, the solution of a metal in an acid—all these different causes of motion may be made to produce the same effect. But in the animal we recognize as the ultimate cause of all force only one cause, the chemical action which the elements of the food and the oxygen of the air mutually exercise on each other. The only known ultimate cause of vital force, either in animals or plants, is a chemical action. He next proceeds to shew that the heat evolved by the combustion of carbon is sufficient to account for the production of animal heat. One ounce of carbon evolves, during its combustion, as much heat as would raise the temperature of 105 ounces of water at 32° to 167°, that is, by 135°; in all therefore 105 times 135° =14207 degrees of heat. Therefore the 13·9 oz. of carbon which are daily converted into carbonic acid in the body of an adult, evolve 13.9×14207=197477·3 degrees of heat. This amount of heat is sufficient to raise the temperature of one ounce of water by that number of degrees, or from 32° to 197509·3°, or to cause 136·8lbs. of water at 32° to boil; or to heat 370lbs. of water to 98·3°, (the precise temperature of the human body); or to convert into vapour 24lbs. of water at 98·3°. And if we assume that the quantity of water vaporized through the skin and lungs amounts to 3lbs., we still have 146380 degrees of heat remaining, which are dissipated by radiation, by heating the exposed air, and in the excrementitious matters. And when we take into account the heat evolved by the hydrogen of the food, and the small specific heat possessed by the organs generally, no doubt can be entertained that the heat evolved in the process of combustion to which the food is subjected in the body, is amply sufficient to explain the constant temperature of the body, as well as the evaporation from the skin and lungs. From what has preceded, it follows that the amount of carbon consumed in food should depend on the climate, density of air, and the occupation of the individual.

The professor, having now discussed the source of animal heat, next proceeds to consider what are the ingredients of the food which may properly be considered to be nutritious—now if we hold that increase of mass in the animal body, the development of its organs and the supply of waste, are all dependent on the blood, those substances only can be considered nutritious, which are capable of conversion into blood. To determine, therefore, what substances are capable of affording nourishment, it is only necessary to ascertain the composition of the food, and to compare it with that of the ingredients of the blood. The chief ingredients of the blood, fibrine and albumen, contain in all seven chemical elements, among which nitrogen, phosphorus, and sulphur are found. They contain also the earth of bones. Chemical analysis has led to the remarkable result that fibrine and albumen contain the same organic elements united in the same proportion; the particles however constituting them are arranged in a different order; but in the ultimate proportion of the organic elements they are identical. Both albumen and fibrine, in the process of nutrition are capable of being converted into muscular fibre, and muscular fibre is capable of being re-converted into blood. If we now compare the composition of all organized parts with that of fibrine and albumen, the following relations present themselves:—All parts of the animal body which have a decided shape, which form parts of organs, contain nitrogen. No part of an organ which possesses motion and life, is destitute of nitrogen; all of them contain likewise carbon and the elements of water, the latter, however in no case in the proportion to form water. The chief ingredients of the blood contain nearly 17 per cent of nitrogen, and no part of an organ contains less that 17 per cent of nitrogen.

It has been proved that the animal body is incapable of producing an elementary body, such as carbon or nitrogen, out of substances which do not contain it; and it follows that all kinds of food fit for the production either of blood, or of cellular tissue, membranes, skin, hair, &c. must contain a certain amount of nitrogen, that element being essential to the composition of the above-named organs, because the organs cannot create it from the other elements presented to them; and finally, because no nitrogen is absorbed from the atmosphere in the vital process. The substance of the brain and nerves contains a large quantity of albumen, besides two peculiar fatty acids, one of which contains nitrogen. Water and common fat are those ingredients of the body which are destitute of nitrogen. Both are unorganized. The inorganic constituents of the body are, iron, lime, magnesia, common salt, and the alkalies. The nutritive process in the carnivora is seen in its simplest form. These animals live on the blood and flesh of the graminivora whose blood and flesh is identical with their own. The nutriment of the carnivorous animals is derived originally from blood; in their stomach it becomes dissolved, and capable of reaching all other parts of the body: in its passage it is again converted into blood, from which blood are reproduced all those parts of their organization, which have undergone change. Thus then, in a chemical sense, a carnivorous animal may be said to consume itself; as that which serves for its nutrition is identical with those parts of its organization which are to be renewed. The professor then enquires from what substances is the blood formed by means of which the organs of the graminivorous animals are developed? The digestive organs in those are less simple and their food consists of vegetables, the great mass of which contains but little nitrogen. The nitrogenized compounds of vegetables forming the food of graminivorous animals are called vegetable fibrine, vegetable albumen, and vegetable caseine. Now analysis has shewn, that they are exactly of the same composition in 100 parts; and, what is still more extraordinary, they are absolutely identical with the chief constituents of the blood—animal fibrine, and animal albumen—they all three dissolve in concentrated muriatic acid with the same deep purple colour, and even in their physical characters there is no difference between animal fibrine and albumen and vegetable fibrine and albumen. By identity, be it remarked, we do not here mean similarity, but an absolute identity, even as far as their organic constituents are concerned. These discoveries admirably exhibit the beauty and simplicity of nutrition in animals. Those vegetable principles which in animals are used to form blood, contain the chief constituents of blood, fibrine and albumen, ready formed, as far as regards their composition. All plants besides contain iron which re-appears in the colouring matter of the blood. Vegetables produce in their organism the blood of all animals; for the carnivora, in consuming the blood and flesh of the graminivora, consume, strictly speaking, only the vegetable principles, which have served for the nutrition of the latter. From all this it follows that the development of the animal organism and its growth are dependant on the reception of certain principles identical with the chief constituents of blood. The animal organism is a higher kind of vegetable, the development of which begins with those substances, with the production of which the life of an ordinary vegetable ends. A very important question still remains to be solved, namely, that of the function performed in the animal system by substances containing no nitrogen, such as sugar, starch, gum, &c. without which the graminivora cannot live; as their food must contain a certain amount of them, and if these compounds are not supplied, death quickly ensues. By a train of peculiarly ingenious reasoning the professor shows that all these substances contain a great excess of carbon, or of carbon and hydrogen, which excess is expended in the production of animal heat, and serves to preserve the organism from the action of atmospheric oxygen.

Some ingenious views are next presented on the uses of the bile in the animal economy. According to many physiologists the bile is only intended for excretion. But quantitative physiology must at once reject the opinion that the bile serves no purpose in the economy, and is incapable of further change.

No part of any organized structure contains soda: it is only met with in the serum of the blood, in the fat of the brain and in the bile. When the compounds of soda in the blood are converted into muscular fibre, membrane, or cellular tissue, the soda they contain must enter into new combinations. The blood which is transformed into organized tissue gives up its soda to the compounds formed by the metamorphoses of the previously existing tissues. In the bile we find one of these compounds of soda. Were the bile intended merely for excretion, we should find it, more or less altered, and also the soda it contains in the solid excrements. But such is not the case—the soda of the bile must, therefore, at all events have returned from the intestinal canal into the organism, and the same must be true of the organic matters combined with it. During the digestive process, therefore, the soda of the bile, and along with it all the soluble parts of that fluid, are returned into the circulation. This soda re-appears in the newly-formed blood, and finally, we find it in the urine in the form of phosphate, carbonate and hippurate of soda.

It cannot be disputed that in an adult carnivorous animal, which neither gains nor loses weight perceptibly from day to day, its nourishment, the waste of organized tissue, and its consumption of oxygen stand to each other in a well-defined and fixed ratio. The carbon of the carbonic acid given off, with that of the urine; the nitrogen of the urine, and the hydrogen given off as ammonia and water; these elements, taken together must be exactly equal in weight to the carbon, nitrogen, and hydrogen of the metamorphosed tissues, and since these last are exactly replaced by the food, to the carbon, nitrogen, and hydrogen of the food. In the young of the carnivora the weight increases perceptibly from day to day. This fact presupposes that the assimilative process in the young animal is more enegetic than the process of transformation in the tissues. Now it is well known that the number of respirations, and consequently the consumption of oxygen is greater in the young than in the adult animal. But since the metamorphosis of organised parts goes on more slowly, there would ensue a deficiency of those substances, the carbon and hydrogen of which are adapted for combination with oxygen. This carbon and hydrogen however, infinite wisdom has supplied to the young animal in its natural food. The carbon and hydrogen of butter, and the carbon of the sugar of milk, no part of which can yield blood, are destined for the support of the respiratory process, at an age when a greater resistance is opposed to the metamorphosis of existing organisms. The butter and sugar of milk are given out in the form of carbonic acid and water, and their conversion into oxidized products furnishes the clearest proofs that far more oxygen is absorbed than is required to convert the carbon and hydrogen of the metamorphosed tissues into carbonic acid and water.

Our author next adverts to the substances which form the principal part of the food of the graminivora. These are found to contain a large proportion of starch, cane sugar, gum, sugar of milk and grape sugar; the great similarity of composition in these different substances, which perform so important a part in the nutritive process of the graminivora, is very striking. For the same number of equivalents of carbon, starch contains 10 equivalents, cane-sugar and gum 11 equivalents, sugar of milk 12 equivalents, and grape sugar 14 equivalents of water, or the elements of water. In these different substances, some one of which is never wanting in the food of the graminivora, there is added to the nitrogenized constituents of this food from which the blood is formed a certain excess of carbon, which the organism cannot employ to produce fibrine or albumen, as the nitrogenized constituents of the food already yield sufficient carbon for that purpose; but the function which these substances (sugar, gum, &c.) perform in the vital process of the graminivora will at once appear evident, if we only take into account the small quantity of carbon which these animals consume in the nitrogenized constituents of their food, a quantity which bears no proportion to the large amount of oxygen absorbed through the skin and lungs. The professor shews that in the nitrogenized constituents of the quantity of food necessary for a horse daily there are contained only about $14\frac{1}{2}$ oz. of carbon for the support of respiration, whereas from the immense quantity of oxygen which this animal takes in, 79 oz. of carbon is the quantity required for daily consumption, this deficiency is added to his food in various forms, as starch, sugar, &c. with which the animal must be supplied, or his organism will be destroyed by the action of the oxygen.

A nation of hunters, restricted to a limited extent of land, would find itself totally unable to increase its population beyond a certain point. The extent of land being limited, the number of animals which it can maintain must be so too, but it is from these the carbon necessary for respiration is to be obtained. Now these animals collect from plants the constituents of their organs and of their blood, and then yield them to the savages who live by the chase alone. They again receive this food, unaccompanied by those non-nitrogenized principles, which contain so much carbon, and which during the life of the animal served to support the respiratory process. In such men confined to animal diet it is the carbon of the flesh and blood, which must take the place of the starch and sugar. Now 15lbs. of flesh contain not more carbon than 4lbs. of starch, and while the savage with one animal and an equal weight of starch could sustain life for a certain number of days, he would be obliged, if restricted to flesh, to consume five such animals, in order to procure the carbon necessary for respiration during the same time. These considerations point out to us the close connexion between agriculture and the multiplication of the human species. The object of agriculture is to produce a maximum of those substances necessary for assimilation and respiration, in the smallest possible space. Grain and other vegetables yield us not only in starch, sugar and gum the carbon so necessary for respiration and the production of animal heat, but also vegetable albumen, fibrine, and caseine, which go to the formation of blood, whence the other parts of our body are developed. We see the laborious exertions, and the great amount of muscular exercise which the savage confined to animal food takes—this he is compelled to do, for the purpose of accelerating the waste of the organized tissues by incessant motion, in order to furnish the matter necessary for respiration. From the difference in the constituents of the urine in the carnivora and graminivora our author shows that the process of metamorphosis in the tissues is different in the two classes, both in form and rapidity. The urine of the carnivora is acid, and contains alkaline bases with uric, phosphoric and sulphuric acids; whilst the urine of the graminivora is alkaline, containing an abundance of alkaline carbonates, and a very minute portion of alkaline phosphates. From this latter circumstance, namely, the very minute quantity of the alkaline phosphates, the professor infers the great slowness with which the tissues in the graminivora are metamorphosed. He states that the phosphates resulting from the change of the tissues re-enter the circulation for the purpose of forming brain and nervous matter, and also the earthy parts of the bones, the organs of excretion not separating these salts from the blood, as in the carnivora.

Our author next considers the relative capacity for increase of mass, and the relative assimilative power in the graminivora and carnivora, and points out a striking difference in both. Carnivorous animals require less food for their mere support, because their skin is destitute of perspiratory pores, and because they lose, for equal bulks, much less heat than graminivorous animals. A spider is observed to suck the blood of the first fly with great voracity, but is not excited by a second: a cat will kill one or perhaps two mice and eat them, but even if she kill a third, she will not eat it. A cow or sheep, in the meadow, eats almost uninterruptedly. Their system possesses the power of converting into organized tissues all the food they devour beyond the quantity required for merely supplying the waste of their bodies. All the excess of blood produced is converted into cellular and muscular tissue; the graminivorous animal becomes fleshy and plump, while the flesh of the carnivorous animal is always tough and sinewy. When the stag, roe-deer or hare, animals which live on the same food as cattle or deer, are well supplied with food, their increase in size will depend on the quantity of vegetable albumen, &c. which they consume. If they take sufficient exercise, enough of oxygen is absorbed to consume the carbon of the gum, sugar, starch, and of all similar constituents of the food. But in the case of domestic or stall-fed animals, more food in the shape of nitrogenized compounds is devoured than is required for reproduction; and at the same time more non-nitrogenized substances are eaten, than is necessary to support respiration and keep up animal heat. From want of exercise these animals absorb much less of oxygen than is required to consume all the carbon; the surplus is employed in forming *fat*. Thus then the formation of fat in the animal body results from a want of due proportion between the food taken into the stomach and the oxygen absorbed by the lungs and skin. From all that precedes we may safely infer that there is a close connexion between non-nitrogenized food, such as starch, gum, sugar, &c. and the production of fat. On comparing the composition of sugar of milk, of starch, and of the other varieties of sugar with that of mutton and beef suet and human fat we find they all agree in the proportion of carbon and hydrogen being the same, and that they differ only in that of oxygen. By comparing the formulæ of starch and of fat, it would appear that the former may pass into the latter by a mere separation of a part of its oxygen. Now it being certain that the herbs and roots consumed by the cow contain no butter; that the hay and other fodder of oxen contains no beef-suet, it must be admitted that the fat found in the bodies of these animals is formed in their organism; whence we may be warranted in concluding that a certain quantity of oxygen, in some form or other, separates from the constituents of their food. The chemical analysis of the constituents of the food of the graminivora shews that they contain carbon and oxygen in certain proportions; which, when reduced to equivalents, yield the following series:—

| | | | | |
|---|---|---|---|---|
| In vegetable fibrine, albumen, and caseine there are contained for | 120 | equiv. carbon | 36 | equiv. oxygen, |
| In starch .. .. .. .. .. .. | 120 | ,, | 100 | ,, |
| In cane sugar .. .. .. .. .. | 120 | ,, | 110 | ,, |
| In gum .. .. .. .. .. .. | 120 | ,, | 110 | ,, |
| In sugar of milk .. .. .. .. | 120 | ,, | 120 | ,, |
| In grape sugar .. .. .. .. .. | 120 | ,, | 140 | ,, |

*Now in all fatty bodies, there are contained, on an average*—for 120 equiv. carbon only 10 equiv. oxygen.

Since the carbon of the fatty constituents of the animal body is derived from the food, it is clear, if we suppose fat to be formed from albumen, fibrine, or caseine, that for every 120 equivalents of carbon deposited as fat, 26 equivalents of oxygen must be separated from the elements of these substances; and, further, if we conceive fat to be formed from starch, sugar, or sugar of milk, there must be separated 90, 100, and 110 equivalents of oxygen from these compounds respectively. Accordingly there is but one way in which the formation of fat in the animal body is possible, and that is precisely the way in which it takes place in plants; it is a separation of oxygen from the elements of the food.

The deposition of fat in the animal body is considered by our author as an abnormal condition, depending on the disproportion between the quantity of carbon in the food and that of oxygen absorbed by the skin and lungs; this deposition is a consequence of a deficient supply of oxygen; oxygen being absolutely indispensable for the dissipation of the excess of carbon. This deposition is never seen in wild animals in a state of nature; neither is it seen in the Bedouin or in the Arab of the desert, who exhibits with pride to the traveller his lean, muscular, sinewy limbs, entirely free from fat; but in prisons and jails, it appears as a puffiness in the inmates, fed, as they are, on a poor and scanty diet. Now though the formation of fat depends on a deficiency of oxygen, still in this process a new source of oxygen is opened and a new cause of animal heat; for the oxygen set free in the formation of fat is given out in combination with carbon and hydrogen in the form of carbonic acid and water, by which heat is generated. Thus, in the formation of fat, the vital force possesses a means of counteracting a deficiency in the supply of oxygen, and consequently in that of the heat indispensable for the vital process.

In some diseases the starch, sugar, &c. of the food do not undergo the changes which enable them to assist respiration, and consequently to be converted into fat. Thus, in diabetes mellitus, the starch is only converted into grape sugar, which is expelled from the body without further change. In other diseases, as in inflammation of the liver, we find the blood loaded with fat and oil; and in the composition of the bile there is nothing inconsistent with the supposition that some of its constituents may be formed into fat.

From the preceding observations it appears that the substances constituting the food of man may be divided into two classes: into *nitrogenized* and *non-nitrogenized.* The former are capable of being converted into blood, whilst the latter are not so. Out of those substances which are adapted to the formation of blood are formed all the organised tissues. The other class of substances, in the normal state of health, serve to support the process of respiration. The former our author calls the *plastic elements of nutrition;* the latter, *elements of respiration.*

Among the former we reckon vegetable fibrine, vegetable albumen, vegetable caseine, animal flesh and animal blood: among the latter are, fat, starch, gum, cane sugar, grape sugar, sugar of milk, pectine, bassorine, wine, beer, spirits. The nitrogenized constituents of vegetable food have a composition identical with that of the constituents of the blood. No nitrogenized compound, whose composition differs from that of fibrine, albumen, and caseine, is capable of sustaining the vital process in animals. The animal organism unquestionably possesses the power of forming, from the constituents of its blood, the substance of its membranes and cellular tissue, of its nerves and brain, &c. But the blood must be supplied to it ready formed in its chemical composition—otherwise a period is soon put to the formation of blood and consequently to life. This explains to us how it happens that the tissues yielding gelatine, are not adapted for the support of the vital process; for their composition is different from that of fibrine or albumen. That is, those parts of the animal organism which form the blood do not possess the power of effecting a transformation in the arrangements of the elements of gelatine, or of the tissues containing it. While in the body of a starving or sick individual, the fat disappears, and the muscular tissue returns to the form of blood, we find the tendons and membranes retain their natural condition. When we consider the transformation of the albumen of the blood into a part of an organ composed of fibrine, the identity of composition in the two substances renders the change easily conceivable. Hence, some physiologists have supposed, not without some reason, that gelatine, when taken in the dissolved state, is again converted in the body, into cellular tissue, membrane and cartilage, that it may serve for the reproduction of such parts of those tissues as have been wasted, and also for their growth. And when the powers of nutrition in the whole body are affected by a change of the health, then, even should the power of forming blood remain the same, the organic force by which the constituents of the blood are changed into cellular tissue and membrane, must be enfeebled by sickness. In the sick man the power to produce the necessary metamorphosis must be impaired as well in the stomach, as in all other parts. In this state experience shews that gelatinous matters in a dissolved state exercise a decided influence on the health. Given in a form adapted for assimilation, they serve to husband the vital force, just as may be done in the case of the stomach, by a judicious preparation of the food.

In the SECOND PART of his work, the subject of which is the *Metamorphosis of Tissues*, Professor Liebig examines in detail the several chemical processes engaged in the formation of bile, of urea, uric acid and its compounds, as also of the cerebral and nervous substance. Previously, however, to entering on these matters, he makes some preparatory remarks on the ultimate organic elements of certain substances, closely connected with his subject. He states, that recent experiment has demonstrated the existence of numerous compounds, both nitrogenised and non-nitrogenised, which, with the greatest diversity in external characters, possess the same composition in 100 parts, many of them containing the same absolute amount of equivalents of each element. Such compounds are designated *isomeric* and *polymeric*.

As an instance, he adduces the absolute identity of composition in the chief constituents of blood and the nitrogenised compounds in vegetable food. Albumen, fibrine and caseine, though differing in external characters, contain exactly the same proportion of organic elements. When animal albumen, fibrine, and caseine are dissolved in a moderately strong solution of caustic potash, and the solution is exposed for some time to a high temperature, these substances are decomposed. The addition of acetic acid to the solution causes, in all three, the separation of a gelatinous translucent precipitate, which has exactly the same character and composition, from whichever of the three substances abovementioned it has been obtained. This compound has been found by Mulder to contain the same organic elements, and exactly the same proportion as the animal matters from which it is prepared. Hence the chief constituents of the blood and the caseine of milk may be considered as compounds of phosphates and other salts, and of sulphur and phosphorus, with a compound of carbon, nitrogen, hydrogen, and oxygen, in which the relative proportion of these elements is invariable; and this compound may be considered as the commencement and starting point of all other animal tissues, because these are all produced from the blood. To this product of the decomposition of albumen, fibrine, &c. by potash, the name of *proteine* has been given, (*πρωτεύω* to hold the first place). The blood, or its constituents, are compounds of this proteine, with various proportions of inorganic substances. It has been ascertained by experiment that vegetable albumen, fibrine, and caseine are acted on by potash in the same way as animal albumen, fibrine, &c. Hence, then, it may be laid down as a law, founded on experience, that vegetables produce, in their organism, compounds of proteine; and that out of these compounds the various tissues and parts of the animal body are developed by the vital force with the aid of the oxygen of the atmosphere, and of the elements of water. The proposition, that all the organic nitrogenised constituents of the body, how different soever they may be in composition, are derived from proteine, being formed from it by the addition or subtraction of the elements of water or oxygen, the author illustrates very happily by referring to the development of the young animal in the egg. The egg contains no other nitrogenised compound except albumen—the albumen of the yolk is identical with that of the white. Yet we see in the process of incubation during which nothing but the oxygen of the air is introduced, that out of the albumen feathers, claws, globules of the blood, fibrine, membrane and cellular tissue, arteries and veins are produced. Consequently the true starting point for all the tissues is albumen, into which all nitrogenised articles of food, whether of an animal or vegetable nature, must be converted, before they can take part in the process of nutrition.

Our author now comes to the function or process of digestion, which he considers to be totally independent of the vital force, and to take place in virtue of a purely chemical action, similar to those processes of decomposition, called putrefaction, fermentation, or decay. To understand this part of our author's work, it will be necessary to take his account of the nature of fermentation or putrefaction; he describes it to be a process of transformation—that is, a new arrangement of the elementary particles or atoms, of a compound, yielding two or more new groups or compounds, and caused by contact with other substances, the elementary particles of which are themselves in a state of transformation or decomposition. It is a communication, or an imparting of a state of motion, which the atoms of a body in a state of motion are capable of producing in other bodies whose elementary particles are held together only by a feeble attraction. Thus the clear gastric juice contains a substance in a state of transformation, by the contact of which with those constituents of the food, which, by themselves, are insoluble in water, the latter acquire, in virtue of a new grouping of their atoms, the property of dissolving in that fluid. During digestion, the gastric juice, when separated, is found to contain a free mineral acid, the presence of which checks all further change. There cannot be a doubt that the substance present in the gastric juice in a state of change is a product of the transformation of the stomach itself. It is known that no substances possess, in so high a degree as those arising from the progressive decomposition of the tissues containing gelatine or chondrine, the property of exciting a change in the arrangement of the elements of other compounds. When the lining membrane of the stomach of any animal, as that of the calf, is cleared by continued washing with water, it produces no effect, when brought into contact with a solution of sugar, with milk, or other substances. But if the same membrane be exposed for some time to the air, or dried, and then placed in contact with such substances, the sugar is changed, according to the state of decomposition of the animal matter, either into lactic acid, into mannite, and mucilage, or into alcohol and carbonic acid; while milk is instantly coagulated. Thus, as in the germination of seeds, the presence of a body in a state of decomposition, or transformation, which has been called *diastase*, effects the solution of the starch—that is, its conversion into sugar; so a product of the metamorphosis of the substance of the stomach, being itself in a state of metamorphosis which is completed in the stomach, effects the solution of all such parts as are capable of assuming a soluble form. Our author here contradicts the opinion entertained by several physiologists, namely, that lactic acid is formed during digestion, and hence that this acid is necessary for the completion of this process. The presence of free muriatic acid in the gastric juice, first noticed by Prout, has been confirmed by all those chemists, who have since examined that fluid. In the action of the gastric juice on the food, no other element takes a share, except the oxygen of the atmosphere, and the elements of water. This oxygen is introduced directly into the stomach in the saliva which possesses the remarkable property of enclosing air in the shape of froth, in a far higher degree than even soap-suds. This air, by means of the saliva, reaches the stomach with the food, and there its oxygen enters into combination, while its nitrogen is given out through the skin and lungs. Rumination, in certain graminivorous animals, has for its object a renewed and repeated introduction of oxygen.

The fact that nitrogen is given out by the skin and lungs, is explained by the property which animal membranes possess of allowing all gases to permeate them. Thus a bladder filled with carbonic acid, nitrogen, or hydrogen gas, if tightly closed and suspended in the air, loses in 24 hours the whole of the enclosed gas; by a kind of exchange it passes out into the atmosphere, while its place is occupied by the atmospherical air. This permeability to gases is a mechanical property common to all animal tissues; and it is found in the same degree in the living as in the dead tissue. As an additional instance that gases possess the property of permeating animal tissues, our author adduces the fatal accidents which so frequently occur in wine countries from the drinking of what is called *feather-white wine*. This poisonous wine is still in a state of fermentation, which is increased by the heat of the stomach. The carbonic acid gas which is disengaged penetrates through the parietes of the stomach, through the diaphragm, and through all the intervening membranes, into the air-cells of the lungs, out of which it displaces the atmospherical air. The patient dies with all the symptoms of asphyxia caused by an irrespirable gas; and the surest proof of the presence of the carbonic acid in the lungs is the fact, that the inhalation of ammonia is recognised as the best antidote against this kind of poisoning.

Just as muscular fibre, when separated from the body, communicates the state of decomposition existing in its elements to the peroxide of hydrogen, so a certain product, arising by means of the vital process, and in consequence of the transposition of the elements of parts of the stomach, and of the other digestive organs, while its own metamorphosis is accomplished in the stomach, acts on the food. The insoluble matters become soluble—they are digested.

As hard-boiled white of egg or fibrine, when rendered soluble by certain liquids, retain all their properties except the solid form without the slightest change, in the same manner, in the digestive process in the healthy state, the food only undergoes a change in its state of cohesion, becoming fluid without any other change of properties.

A strong argument adduced by our author in favour of the analogy between digestion and fermentation or putrefaction, is, that all substances capable of arresting the latter processes in liquids, also arrest digestion, when taken into the stomach. The action of empyreumatic matters in coffee and tobacco-smoke, of creosote, of mercurials, &c. &c. deserves on this account peculiar attention with reference to dietetics.

.......... The formula $C_{48}\ H_{36}\ N_6\ O_{14}$ is that which most accurately expresses the composition of proteine, or the relative proportions of the organic elements in the blood, as ascertained by analysis. Albumen, fibrine, and caseine contain proteine; caseine contains, besides, sulphur, but no phosphorus; albumen and fibrine contain both these substances chemically combined—the former more sulphur than the latter.......

If we reflect that from the albumen and fibrine of the body all the other tissues are derived, it is perfectly clear, that this can only occur in two ways. Either certain elements have been added to, or removed from, their constituent parts. If we now look, for example, for an analytical expression of the composition of cellular tissue, of the tissues yielding gelatine, of tendons, of hair, of horn, &c. in which the number of atoms of carbon is made invariably the same as in albumen and fibrine, we can then see, at the first glance, in what way the proportion of the other elements has been altered; but this includes all that physiology requires in order to obtain an insight into the true nature of the formative and nutritive processes in the animal body.

From the researches of Mulder and Scherer we obtain the following empirical formulæ: (P phosphorus—S sulphur.)

*Composition of Organic Tissues.*

| | |
|---|---|
| Albumen . . . . | $C_{48}N_6\ H_{36}O_{14}+P+S$ |
| Fibrine . . . . | $C_{48}N_6\ H_{36}O_{14}+P+2S$ |
| Caseine . . . . | $C_{48}N_6\ H_{36}O_{14}+S$ |
| Gelatinous tissues, tendons . | $C_{48}N_{7 \cdot 5}H_{41}O_{18}$ |
| Chondrine . . . . | $C_{48}N_6\ H_{40}O_{20}$ |

| | |
|---|---|
| Hair, horn . . . . . | $C_{48}N_7 H_{39}O_{17}$ |
| Arterial membrane . . . | $C_{48}N_6 H_{38}O_{16}$ |

The composition of these formulæ shews, that when proteine passes into chondrine (the substance of the cartilage of the ribs), the elements of water, with oxygen, have been added to it; while in the formation of the serous membranes, nitrogen also has entered into combination.

If we represent the formula for proteine by Pr, then nitrogen, hydrogen, and oxygen have been added to it in the form of known compounds, and in the following proportions, in forming the gelatinous tissues, hair, horn, arterial membrane, &c.

| | Proteine. | Ammonia. | Water. | Oxygen. |
|---|---|---|---|---|
| Fibrine, albumen . . . | Pr. | | | |
| Arterial membrane . . | Pr. | | + 2HO | |
| Chondrine . . . . . | Pr. | | + 4HO | + 2O |
| Hair, horn . . . . | Pr. | + NH | + HO | + 3O |
| Gelatinous tissues . . . | 2Pr. | + 3NH | + HO | + 7O |

From what precedes, it appears that all the tissues of the body contain, for the same amount of carbon, more oxygen than the constituents of blood. During their formation, oxygen, either from the atmosphere or from the elements of water, has been added to the elements of proteine. In hair and gelatinous membrane we observe, further, an excess of nitrogen and hydrogen, and that in the proportions to form ammonia.

The above formulæ express with precision the differences of composition in the chief constituents of the animal body: they shew that for the same amount of carbon the proportion of the other elements varies, and how much more oxygen or nitrogen one compound contains than another. By means of these formulæ we can trace the production of the different compounds from the constituents of the blood. For the same amount of carbon gelatinous membranes and tissues contain more nitrogen, oxygen, and hydrogen than proteine: they may be formed from albumen by the addition of oxygen, of the elements of water, and of those of ammonia, accompanied by the separation of sulphur and phosphorus. That gelatinous tissues contain no proteine is proved by the action of caustic alkalies on them. Gelatinous tissue, though formed from compounds of proteine, no longer belong to the series of the compounds of proteine. It is a fact deduced from observation that nature has exclusively destined compounds of proteine for the production of blood. No substance analogous to the gelatinous tissue is found in vegetables. This tissue is not a compound of proteine; it contains neither sulphur nor phosphorus, and it contains more nitrogen or less carbon than proteine. The compounds of proteine under the influence of the vital energy of the organs which form the blood, assume a new form, but are not altered in composition; while these organs do not possess the power of producing compounds of proteine out of substances which contain no proteine. Animals fed exclusively with gelatine, died with the symptoms of starvation; gelatinous tissue is, in fact, incapable of conversion into blood.

Our author now proceeds to develope analytically the principal metamorphoses which occur in the animal body.

If, he says, it be true that all parts of the body are formed and developed from the blood or the constituents of the blood, that the existing organs at every moment of life are transformed into new compounds under the influence of the oxygen introduced in the blood, then the animal secretions must of necessity contain the products of the metamorphosis of the tissues.——If it be further true that the urine contains those products of metamorphosis which contain the most nitrogen, and the bile those which are richest in carbon, from all the tissues which in the vital process have been transformed into unorganized compounds, it is clear that the elements of the bile and of the urine, added together, must be equal, in the relative proportion of these elements, to the composition of the blood. The organs are formed from the blood and contain the elements of the blood; they become transformed into new compounds, with the addition only of oxygen and water. Hence the relative proportion of carbon and nitrogen must be the same as in the blood. If then we subtract from the composition of blood the elements of the urine, the remainder, deducting the oxygen and water which have been added, must give the composition of the bile—or, if from the elements of the blood, we subtract the elements of the bile, the remainder must give the composition of urate of ammonia, or of urea and carbonic acid. This mode of viewing the subject has led to the true formula of bile.—The analyses of Playfair and Boeckman gave for flesh, and for blood, one and the same formula, namely, $C_{48} N_6 H_{39} O_{15}$. The chief constituent of bile is a compound, according to Demarçay, analogous to soaps, of soda with a peculiar substance, named *choleic acid.* This acid is resolved by the action of muriatic acid, into *ammonia, taurine,* and a new acid, *choloidic acid,* which contains no nitrogen. And when boiled with caustic potash, choleic acid is resolved into *carbonic acid, ammonia,* and another new acid, *cholic acid.* Professor Liebig here assumes a formula of choleic acid, and by deducting from the elements of this acid, the elements of ammonia and taurine, he obtains the formula of choloidic acid. Again, by deducting from the elements of the same choleic acid the elements of urea and two atoms of water, the composition of cholic acid will remain. The formulæ so obtained of choloidic acid and of cholic acid coinciding with the results of analysis, the assumed formula of choleic acid may be deemed accurate. Again by adding half the numbers representing the formula of choleic acid to the elements of the urine of the serpent—the neutral urate of ammonia, the result turns out to be identical with the formula expressing the composition of the blood, with the addition of one equivalent of oxygen and one of water. Again, by adding to the elements of proteine those of three equivalents of water, we obtain precisely the same formula as that already obtained by adding together choleic acid and urate of ammonia, differing only by one equivalent of hydrogen. If, then, we consider choleic acid and urate of ammonia the products of the transformation of muscular fibre, since no other tissue in the body contains proteine, there exists in fibrine, with the addition of the elements of water, all the elements essential to this metamorphosis. This form of metamorphosis is applicable to the vital transformations in the lower classes of amphibia. In the higher classes of animals the uric acid disappears in the urine, and is replaced by urea. This disappearance of uric acid and the production of urea depend on the amount of oxygen absorbed in respiration, and on the quantity of water consumed by different animals in a given time. When uric acid is subjected to the action of oxygen, it is first resolved into alloxan and urea. A new supply of oxygen acting on the alloxan causes it to resolve itself either into oxalic acid and urea, into oxaluric and parabonic acid, or into carbonic acid and urea. In mulberry calculi we find oxalate of lime, in other calculi urate of ammonia, and always in persons, in whom, from want of exercise and labour, or from other causes, the supply of oxygen has been diminished. Calculi containing uric acid or oxalic acid are never found in phthisical patients; and it is a common occurrence in France, among patients suffering from calculous complaints that when they go to the country, where they take more exercise, the compounds of uric acid, which were deposited in the bladder during their residence in town, are succeeded by oxalates (mulberry calculus) in consequence of the increased supply of oxygen. With a still greater supply of oxygen they would have yielded, in healthy subjects, only the last product of the oxidation of uric acid, namely carbonic acid and urea.

From the undoubted fact that all substances incapable of further use in the system are separated by the kidneys and expelled from the body in the urine, altered or unaltered, it has been erroneously inferred that the food, and especially nitrogenised food, may have a direct influence on the formation of urinary calculi—such an idea is unfounded. Boiled and roasted flesh is at once converted into blood; while the uric acid and urea are derived from the metamorphosed tissues. The quantity of the latter increases with the rapidity of transformation in a given time, but bears no proportion to the amount of food taken in the same time. In a starving man making much exertion more urea is secreted than in the most highly-fed individual in a state of rest. In fevers and during rapid emaciation the urine contains more urea than in the state of health. The uric acid in the urine of man disappears, when he receives through the skin and lungs a quantity of oxygen sufficient to oxidize the products of the transformation of the tissues. The use of wine and fat, which, when taken into the organism, undergo no other change but that of combining with oxygen, has a marked influence on the formation of uric acid. The urine, after the use of fat food, becomes turbid, and deposits minute crystals of uric acid. The same thing happens after the use of wines in which the alkali necessary to hold the uric acid in solution is wanting, but never from the use of Rhenish wines, which contain so much tartar. In animals which drink much water, by which the sparingly soluble uric acid is kept dissolved, so that the inspired oxygen can act on it, no uric acid is found in the urine, but only urea.

If to one atom of uric acid we add six atoms of oxygen and four atoms of water, it resolves itself into urea and carbonic acid. The urine of the herbivora contains no uric acid, but ammonia, urea, and hippuric or benzoic acid.

Professor Liebig next observes that if to the formula of proteine, multiplied by 3, we add the elements of 4 atoms of water, and if we deduct from the sum of all the elements half of the elements of choloidic acid, there remains a formula which expresses very nearly the composition of gelatine:—and if from this formula of gelatine the elements of 2 atoms of proteine be subtracted, there remain the elements of urea, uric acid and water, or of three atoms of allantoine and three atoms of water. Assuming the correctness of this formula, it then appears that the elements of two atoms of proteine *plus* the nitrogenised products of the transformation of a third atom of proteine (uric acid and urea) and water; or three atoms of proteine, *minus* the elements of a compound containing no nitrogen, which actually occurs as one of the products of the transformation of chloric acid, yield in both cases a formula closely approaching the composition of gelatinous tissues: that is, in other words, gelatinous tissue may be produced by the addition, to the elements of proteine, of allantoine and water, or of water, urea and uric acid; or by the separation from the elements of proteine of a compound containing no nitrogen. No other constituent of the bile has as yet been taken into the calculation besides choleic acid; because it alone is known with certainty to contain nitrogen. Now if it be admitted that its nitrogen is derived from the metamorphosed tissues, it is probable that the carbon, and other elements which it contains, are derived from the same source.

The author here cautions us against supposing that the nitrogen of the food can pass into the urine as urea, without having previously become part of an organized tissue; the untenableness of such a supposition will at once appear, when we recollect that the albumen, from which such nitrogen should be derived, suffers no change in passing through the liver or kidneys; so that these organs cannot be adapted for the alteration or decomposition of the substance from which all the other organs of the body are to be formed.

To the question, what becomes in man of the compounds of proteine taken in excess, what change is undergone by the superabundant nitrogenised food? he answers, the blood-vessels become distended with excess of blood, the other vessels with excess of their fluids; and if the too great supply of food be kept up, and the blood or other fluids adapted for forming blood be not applied to their natural purposes, if the soluble matters be not taken up by the proper organs, various gases are disengaged, as in processes of putrefaction, the excrements assume an altered quality in colour, smell, &c. Now none of these effects should occur if the liver and kidneys were capable of effecting the resolution of the superabundant compounds of proteine into urea, uric acid, and bile. All the observations which have been made in reference to the influence of nitrogenised food on the composition of the urine have entirely failed to demonstrate the existence of any direct influence of the kind: the phenomena are capable of a much simpler interpretation, if, along with the food, we consider the mode of life and habits of the individuals who have been the subjects of investigation. Gravel and calculus occur in persons who use very little animal food. Concretions of uric acid have never been observed in carnivorous mammalia, living in the wild state, and among nations which live entirely on flesh, deposits of uric acid concretions in the limbs or in the bladder are utterly unknown. That which must be considered as undeniably true with respect to the origin of bile in the carnivora, cannot hold in regard to all the constituents of the bile secreted by the liver in the herbivora: for with the enormous quantity of bile produced, for instance, by the liver of an ox, it is absolutely impossible to suppose that all its carbon is derived from the metamorphosed tissues. Hence it follows that other substances, besides compounds of proteine, must inevitably take part in forming bile in the herbivora; and these substances can only be the non-nitrogenised constituents of their food.

The consideration of the quantitative proportion of the bile secreted in the herbivora leads to the following conclusions:—

The chief constituents of the bile of the herbivora contain nitrogen, and this nitrogen is derived from compounds of proteine. The bile of this class of animals contains more carbon than corresponds to the quantity of nitrogenised food taken, or to the portion of tissue that has undergone metamorphosis in the vital process. A part of this carbon must, therefore, be derived from the non-nitrogenised parts of the food (starch, sugar, &c.); and in order to be converted into a nitrogenised constituent of bile, a part of these bodies must have combined with a nitrogenised compound derived from a compound of proteine. With respect to this conclusion it is quite indifferent whether that compound of proteine be derived from the food or from the tissues of the body.

The comparison of the amount of carbon in the bile secreted by an herbivorous animal, with the quantity of carbon of its tissues, or of the nitrogenised constituents of its food, which in consequence of the constant transformations may pass into bile, indicates a very striking difference. The carbon of the bile secreted amounts, at least, to more than five times the quantity of that which could reach the liver in consequence of the change of matter in the body, either from the metamorphosed tissues or from the nitrogenised constituents of the food; and we may regard as well-founded the supposition that the non-azotised constituents of the food take a decided share in the production of bile in the herbivora; for neither experience nor observation contradicts the opinion.

Chemical analysis and the study of the living animal body mutually support each other; and both lead to the conclusion that a certain portion of the carbon of the non-azotised constituents of food (of starch, &c., the elements of respiration) is secreted by the liver in the form of bile; and further, that the nitrogenised products of the transformation of tissues in the herbivora do not, as in the carnivora, reach the kidneys immediately or directly, but that, before their expulsion from the body in the form of urine, they take a share in certain other processes, especially in the formation of bile. They are conveyed to the liver with the non-azotised constituents of the food; they are returned to the circulation in the form of bile, and are not expelled by the kidneys till they have thus served for the production of the most important of the substances employed in respiration.

When the urine is left to itself, the urea which it contains is converted into carbonate of ammonia; the elements of urea are in such proportion that, by the addition of the elements of water, all its carbon is converted into carbonic acid, and all its nitrogen into ammonia. * * * * *

The presence of free muriatic acid in the stomach, and that of soda in the blood, prove beyond all doubt the necessity of common salt for the organic processes; but the quantities of soda required by animals of different classes, to support the vital processes, are singularly unequal. If we suppose that a given amount of blood, considered as a compound of soda, passes in the body of a carnivorous animal, in consequence of the change of matter, into a new compound of soda, namely, the bile, we must assume that, in the normal condition of health, the proportion of soda in the blood is amply sufficient to form bile with the products of transformation. The soda which has been used in the vital processes, and any excess of soda, must be expelled in the form of a salt, after being separated from the blood by the kidneys.

Now if it be true, that, in the body of an herbivorous animal, a much larger quantity of bile is produced than corresponds to the amount of blood formed or transformed in the vital processes; if the greater part of the bile, in this case, proceeds from the non-azotised constituents of the food, then the soda of the blood which has been formed into organised tissue (assimilated or metamorphosed) cannot possibly suffice for the daily secretion of bile. The soda, therefore, of the bile of the herbivora must be supplied directly in the food; their organism must possess the power of applying directly to the formation of bile all the compounds of soda present in the food, and decomposable by the organic process. All the soda of the animal body obviously proceeds from the food; but the food of the carnivora contains, at most, only the amount of soda necessary to the formation of blood; and, in most cases, among animals of this class, we may assume that only as much soda as corresponds to the proportion employed to form the blood is expelled in the urine.

When the carnivora obtain in their food as much soda as suffices for the production of their blood, an equal amount is excreted in the urine; when the food contains less, a part of that which would otherwise be excreted is retained by the organism. All these statements are most unequivocally confirmed by the composition of the urine in these different classes of animals. As the ultimate product of the changes of all compounds of soda in the animal body, we find in the urine the soda in the form of a salt, and the nitrogen in that of ammonia or urea. The soda in the urine of the carnivora is found in combination with sulphuric and phosphoric acids; and along with the sulphate and phosphate of soda we never fail to find a certain quantity of a salt of ammonia, either muriate or phosphate of ammonia. There can be no more decisive evidence in favour of the opinion, that the soda of their bile or of the metamorphosed constituents of their blood is very far from sufficing to neutralize the acids which are separated, than the presence of ammonia in their urine. This urine, moreover, has an acid reaction. In contradistinction to this, we find, in the urine of the herbivora, soda in predominating quantity, and that not combined with sulphuric or phosphoric acids, but with carbonic, benzoic, or hippuric acids. These well-established facts demonstrate that the herbivora consume a far larger quantity of soda than is required merely for the supply of the daily consumption of blood. In their food are united all the conditions for the production of a second compound of soda, destined for the support of the respiratory process; and it can only be a very limited knowledge of the vast wisdom displayed in the arrangements of organized nature which can look on the presence of so much soda in the food and in the urine of the herbivora as accidental.

It cannot be accidental that the life, the development of a plant, is dependent on the presence of the alkalies which it extracts from the soil.

This plant serves as food to an extensive class of animals, and in these animals the vital process is again most closely connected with the presence of these alkalies. We find the alkalies in the bile, and their presence in the animal body, is the indispensable condition for the production of the first food of the young animal; for without an abundant supply of potash, the production of milk becomes impossible.

All observation leads, as appears from the preceding exposition, to the opinion, that certain non-azotised constituents of the food of the herbivora (starch, sugar, gum, &c.) acquire the form of a compound of soda, which in their bodies serves for the same purpose as that which we know certainly to be served by the bile (the most highly-carbonized product of the transformation of their tissues) in the bodies of the carnivora. These substances are employed to support certain vital actions, and are finally consumed in the generation of animal heat, and in furnishing means of resistance to the action of the atmosphere. In the carnivora the rapid transformation of their tissues is a condition of their existence, because it is only as the result of the change of matter in the body that these substances can be formed, which are destined to enter into combination with the oxygen of the air; and in this sense we may say that the non-azotised constituents of the food of the herbivora impede the change of matter, or retard it, and render unnecessary, at all events, so rapid a process as occurs in the carnivora.

The quantity of azotised matter, proportionally so small, which the herbivora require to support their vital functions, is closely connected with the power possessed by the non-azotised parts of their food to act as means of supporting the respiratory process; and this consideration seems to render it not improbable, that the necessity for more complex organs of digestion in the herbivora is rather owing to the difficulty of rendering soluble and available for the vital processes certain non-azotised compounds (gum? amylaceous fibre?) than to any thing in the change or transformation of vegetable fibrine, albumen, and caseine into blood; since, for this latter purpose, the less complex digestive apparatus of the carnivora is amply sufficient.

If in man, when fed on a mixed diet, starch performs a similar part to that which it plays in the body of the herbivora; if it be assumed that the elements of starch are equally necessary to the formation of the bile in man as in those animals; then it follows that a part of the azotised products of the transformation of the tissues in the human body, before they are expelled through the bladder, returns into the circulation from the liver in the shape of bile, and is separated by the kidneys from the blood, as the ultimate product of the respiratory process.

When there is a deficiency of non-azotised matter in the food of man, this form of the production of bile is rendered impossible. In that case the secretions must possess a different composition; and the appearance of uric acid in the urine, the deposition of uric acid in the joints and in the bladder, as well as the influence which an excess of animal food (which must be considered equivalent to a deficiency of starch, &c.) exercises on the separation of uric acid in certain individuals, may be explained on this principle. If starch, sugar, &c. be deficient, then a part of the azotised compounds formed during the change of matter will either remain in the situation where they have been formed, in which case they will not be sent from the liver into the circulation, and therefore will not undergo the final changes dependent on the action of oxygen; or they will be separated by the kidneys in some form different from the normal one.

We have here endeavoured to prove that the non-azotised constituents of food exercise a most decided influence on the nature and quality of the animal secretions. Whether this occur directly; whether, that is to say, their elements take a decided share in the act of transformation of tissues; or whether their share in that process be an indirect one, is a question probably capable of being resolved by careful and cautious experiment and observation. It is possible that the non-azotised constituents of food, after undergoing some change, are carried from the intestinal canal directly to the liver, and that they are converted into bile in this organ, where they meet with the products of the metamorphosed tissues, and subsequently complete their course through the circulation.

This opinion appears more probable, when we reflect that as yet no trace of starch or sugar has been detected in arterial blood, not even in animals which had been fed exclusively with these substances. We cannot ascribe to these substances, since they are wanting in arterial blood, any share in the nutritive process; and the occurrence of sugar in the urine of those affected with diabetes mellitus (which sugar, according to the best observations is derived from the food) coupled with its total absence in the blood of the same patients, obviously proves that starch and sugar are not, as such, taken into the circulation.

The writings of physiologists contain many proofs of the presence of certain constituents of the bile in the blood of man in the state of health, although their quantity can hardly be determined. Indeed, if we suppose $8\frac{1}{4}$ ℔. (58,000 grains) of blood to pass through the liver every minute, and if from this quantity of blood 2 drops of bile (3 grs. to the drop) are secreted, this would amount to $\frac{1}{9600}$th part of the weight of the blood, a proportion far too small to be quantitatively ascertained by analysis. The greater part of the bile in the body of the herbivora, and in that of man fed on mixed food, appears from the preceding considerations to be derived from the elements of the non-azotised food. But its formation is impossible without the presence of an azotised body, for the bile is a compound of nitrogen. All varieties of bile yet examined yield, when subjected to dry distillation, ammonia and other nitrogenised products. Taurine and ammonia may readily be extracted from ox-bile; and the only reason why we cannot positively prove that the same products may be obtained from the bile of other animals is this, that it is not easy to procure, in the case of many of these animals, a sufficient quantity of bile for the experiment.

Now whether the nitrogenised compound which unites with the elements of starch to form bile be derived from the food, or from the substance of the metamorphosed tissues, the conclusion that its presence is an essential condition for the secretion of bile cannot be considered doubtful. Since the herbivora obtain in their food only such nitrogenised compounds as are identical in composition with the constituents of their blood, it is at all events clear, that the nitrogenised compound which enters into the composition of bile is derived from a compound of proteine. It is either formed in consequence of a change which the compounds of proteine in the food have undergone, or it is produced from the blood or from the substance of the tissues by the act of their metamorphosis. If the conclusion be accurate, that nitrogenised compounds, whether derived from the blood or from the food, take a decided share in the formation of the secretions, and particularly of the bile, then it is plain that the organism must possess the power of causing foreign matters, which are neither parts nor constituents of the organs in which vital activity resides, to serve for certain vital processes. All nitrogenised substances capable of being rendered soluble, without exception, when introduced into the organs of circulation or of digestion, must, if their composition be adapted for such purposes, be employed by the organism in the same manner as the nitrogenised products which are formed in the act of metamorphosis of the tissues. We are acquainted with several substances, which exercise a most marked influence on the act of transformation as well as on the nutritive process, while their elements take no share in the resulting changes. These are uniformly substances the particles of which are in a certain state of motion or decomposition, which state is communicated to all such parts of the organism as are capable of undergoing a similar transformation. Medicinal and poisonous substances form a second and most extensive class of compounds the elements of which are capable of a direct or an indirect share in the processes of secretion and of transformation. These may be divided into three great orders: the first (including the metallic poisons) consists of substances which enter into chemical combination with certain parts or constituents of the body, while the vital force is insufficient to destroy the compounds thus formed. The second division, consisting of the essential oils, camphor, empyreumatic substances, and antiseptics, &c., possesses the property of impeding or retarding those kinds of transformation to which certain very complex organic molecules are liable; transformations which, when they take place out of the body, are usually designated by the names of fermentation and putrefaction.

The third division of medicinal substances is composed of bodies, the elements of which take a direct share in the changes going on in the animal body. When introduced into the system they augment the energy of the vital activity of one or more organs—they excite morbid phenomena in the healthy body. All of them produce a marked effect in a comparatively small dose, and many are poisonous when administered in larger quantity. None of the substances in this class can be said to take a decided share in the nutritive process or to be employed by the organism in the production of blood; partly because their composition is different from that of blood, and partly because the proportion in which they must be given, to exert their influence, is as nothing, compared with the mass of blood. These substances when taken into the circulation, alter, as is commonly said, the quality of the blood, and in order that they may pass from the stomach into the circulation with their entire efficacy, we must assume that their composition is not affected by the organic influence of the stomach. If insoluble when given, they are rendered soluble in that organ, but they are not decomposed; otherwise they would be incapable of exerting any influence on the blood.

The blood, in its normal state, possesses two qualities closely related to each other, although we may conceive one of them to be quite independent of the other. The blood contains in the form of the globules, the carriers, as it were, of the oxygen which serves for the production of certain tissues, as well as for the generation of animal heat. The globules of the blood, by means of the property they possess of giving off the oxygen they have taken up in the lungs, without losing their peculiar character, determine generally the change of matter in the body. The second quality of the blood, namely, the property which it possesses of becoming part of an organised tissue, and its consequent adaptation to promote the formation and the growth of organs, as well as to the reproduction and supply of water in the tissues, is owing, chiefly, to the presence of dissolved fibrine and albumen. These two chief constituents which serve for nutrition and the reproduction of matter, in passing through the lungs are saturated with oxygen, or, at all events, absorb so much from the atmosphere as entirely to lose the power of extracting oxygen from the other substances present in the blood. We know for certain that the globules of the venous blood, when they come in contact with air in the lungs, change their colour, and that this change of colour is accompanied by an absorption of oxygen; and that all those constituents of the blood, which possess in any degree the power of combining with oxygen, absorb it in the lungs, and become saturated with it. Although in contact with these other compounds, the globules, when arterialised, retain their florid, red colour in the most minute ramifications of the arteries; and we observe them to change their colour, and to assume the dark red colour which characterizes venous blood, only during their passage through the capillaries. From these facts we must conclude that the constituents of arterial blood are altogether destitute of the power to deprive the arterialized globules of the oxygen which they have absorbed from the air; and we can draw no other conclusion from the change of colour which occurs in the capillaries than that the arterialized globules, during their passage through the capillaries, return to the condition which characterized them in venous blood; that, consequently, they give up the oxygen absorbed in the lungs, and thus acquire the power of combining with that element afresh.

We find, therefore, in arterial blood, albumen, which, like all the other constituents of that fluid, has become saturated with oxygen in its passage through the lungs, and oxygen gas, which is conveyed to every particle in the body in chemical combination with the globules of the blood. As far as our observations extend (in the development of the chick during incubation) all the conditions seem to be here united which are necessary to the formation of every kind of tissue; while that portion of oxygen which is not consumed in the growth or reproduction of organs combines with the substance of the living parts, and produces, by its union with their elements, the act of transformation which we have called the change of matter. It is obvious that all compounds, of whatever kind, which are present in the capillaries, whether separated there, or introduced by endosmosis or imbibition, if not altogether incapable of uniting with oxygen, must, when in contact with the arterialised globules, the carriers of oxygen, be affected exactly in the same way as the solids forming parts of living organs. These compounds, or their elements, will enter into combination with oxygen, and in this case there will either be no change of matter, or that change will exhibit itself in another form, yielding products of a different kind. The conception, then, of a change in the two qualities of the blood above alluded to, by means of a foreign body contained in the blood, or introduced into the circulation (a medicinal agent), presupposes two kinds of operation. Assuming that the remedy cannot enter into any such chemical union with the constituents of the blood, as puts an end to the vital activity; assuming, further, that it is not in a condition of transformation capable of being communicated to the constituents of the blood or of the organs, and of continuing in them; assuming, lastly, that it is incapable, by its contact with the living parts, of putting a stop to the change of matter, the transformation of their elements; then, in order to discover the modus operandi of this class of medicinal agents, nothing is left but to conclude, that their elements take a share in the formation of certain constituents of the living body, or in the production of certain secretions.

The vital process of secretion, in so far as it is related to the chemical forces, has been subjected to examination in the preceding observations. In the carnivora, we have reason to believe, that without the addition of any foreign matter in the food, the bile and the constituents of the urine are formed in those parts where the change of matter takes place. In other classes of animals, on the other hand, we may suppose that in the organ of secretion itself, the secreted fluid is produced from certain matters conveyed to it; in the herbivora, for example, the bile is formed from the elements of starch along with those of a nitrogenised product of the metamorphosis of the tissues. But this supposition by no means excludes the opinion, that in the carnivora the products of the metamorphosed tissues are resolved into bile, uric acid, or urea, only after reaching the secreting organ; nor the opinion that the elements of the non-azotised food, conveyed directly by the circulation to every part of the body, where change of matter is going on, may then unite with the elements of the metamorphosed tissues, to form the constituents of the bile and of the urine.

If we now assume that certain medicinal agents may become constituents of secretions, this can only occur in two ways. Either they enter the circulation, and take a direct share in the change of matter, in so far as their elements enter into the composition of the new products; or they are conveyed to the organs of secretion, where they exert an influence on the formation or on the quality of a secretion by the addition of their elements. In either case they must lose in the organism their chemical character; and we know with sufficient certainty that this class of medicinal bodies disappears in the body without leaving a trace. In fact, if we ascribe to them any effect, they cannot lose their peculiar character by the action of the stomach; their disappearance therefore presupposes that they have been applied to certain purposes, which cannot be imagined to occur without a change in their composition. Now, however limited may be our knowledge of the composition of the different secretions, with the exception of the bile, this much is certain, that all the secretions contain nitrogen chemically combined. They pass into fetid putrefaction, and yield either in this change, or by dry distillation, ammoniacal products. Even the saliva, when acted on by caustic potash, disengages ammonia freely.—Medicinal agents may be divided into two classes, the nitrogenised and the non-nitrogenised. The nitrogenised vegetable principles, whose composition differs from that of the proper nitrogenised elements of nutrition, also produced by a vegetable organism, are distinguished beyond all others, for their powerful action on the animal economy. The effects of these substances are singularly varied; from the mildest form of the action of aloes, to the most terrible poison, strychnia, we observe an endless variety of different actions. With the exception of three, all these substances produce disease in the healthy organism, and are poisonous in certain doses. Most of them are alkaline. No remedy, devoid of nitrogen, possesses a poisonous action in a similar dose. The medicinal or poisonous action of the nitrogenised vegetable principles has a fixed relation to their composition—it cannot be supposed to be independent of the nitrogen they contain, but is certainly not in direct proportion to the quantity of nitrogen. Solanine and picrotoxine, which contain least nitrogen, are powerful poisons. Quinine contains more nitrogen than morphia. Caffeine and theobromine, the most highly nitrogenised of all vegetable principles, are not poisonous. A nitrogenised body, which exerts, by means of its elements, an influence on the formation or on the quality of a secretion, must, in regard to its chemical character, be capable of taking the same share as the nitrogenised products of the animal body do in the formation of the bile; that is, it must play the same part as a product of the vital process. On the other hand, a non-azotised medicinal agent, in so far as its action affects the secretions, must be capable of performing in the animal body the same part as that which we have ascribed in the formation of bile, to the non-azotised elements of food.

Thus, if we suppose that the elements of hippuric and uric acids are derived from the substance of the organs in which vitality resides; that, as products of the transformation of these organs, they lose the vital character, without losing the capacity of undergoing changes under the influence of the inspired oxygen, or of the apparatus of secretion; we can hardly doubt that similar nitrogenised compounds, products of the vital process in plants, when introduced into the animal body, may be employed by the organism exactly in the same way as the nitrogenised products of the metamorphosis of the animal tissues themselves. If hippuric or uric acids, or any of their elements, can take a share, for example, in the formation and supply of bile, we must allow the same power to other analogous nitrogenised compounds.

We shall certainly never be able to discover how men were led to the use of the hot infusion of the leaves of a certain shrub (tea) or of a decoction of certain roasted seeds (coffee). Some cause there must be which would explain how the practice has become a necessary of life to whole nations. But it is surely still more remarkable, that the beneficial effects of both plants on the health must be ascribed to one and the same substance, the presence of which in two vegetables, belonging to different natural families, and the produce of different quarters of the globe, could hardly have presented itself to the boldest imagination. Yet recent researches have shewn, in such a manner as to exclude all doubt, that caffeine, the peculiar principle of coffee, and theine, that of tea, are, in all respects, identical.

It is not less worthy of notice, that the American Indian, living entirely on flesh, discovered for himself, in tobacco-smoke, a means of retarding the change of matter in the tissues of his body, and thereby of making hunger more endurable; and that he cannot withstand the action of brandy, which, acting as an element of respiration, puts a stop to the change of matter by performing the function which properly belongs to the products of the metamorphosed tissues. Tea and coffee were originally met with among nations whose diet is chiefly vegetable. Without entering minutely into the medicinal action of caffeine (theine), it cannot but appear a most striking fact, even if we were to deny its influence on the process of secretion, that this substance, with the addition of oxygen and the elements of water, can yield taurine, the nitrogenised compound peculiar to bile. A similar relation exists in the case of the peculiar principle of asparagus, and of althœa, asparagine; which also by the addition of oxygen and the elements of water, yields the elements of taurine. The addition of the elements of water and of a certain quantity of oxygen to the elements of theobromine, the characteristic principle of the cacao bean (theobroma cacao), yields the elements of taurine and urea, of taurine, carbonic acid and ammonia, or of taurine and uric acid. To see how the action of caffeine, asparagine, theobromine, &c. may be explained, we must call to mind that the chief constituent of the bile contains only 3·8 per cent. of nitrogen, of which only the half, or 1·9 per cent. belongs to the taurine. Bile contains, in its natural state, water and solid matter, in the proportion of 90 parts by weight of the former to 10 of the latter. If we suppose these 10 parts by weight of solid matter to be choleic acid, with 3·87 per cent. of nitrogen, then 100 parts of fresh bile will contain 0·171 parts of nitrogen in the shape of taurine. Now this quantity is contained in 0·6 parts of caffeine; or $2\frac{8}{10}$ths grains of caffeine can give to an ounce of bile the nitrogen it contains in the form of taurine. If an infusion of tea contain no more than the $\frac{1}{10}$th of a grain of caffeine, still if it contribute, in point of fact, to the formation of bile, the action, even of such a quantity, cannot be looked on as a nullity. Neither can it be denied that in the case of the excess of non-azotised food and a deficiency of motion, which is required to cause a change of matter in the tissues, and thus to yield the nitrogenised product, which enters into the composition of the bile; that in such a condition, the health may be benefited by the use of compounds which are capable of supplying the place of the nitrogenised product produced in the healthy state of the body, and essential to the production of an important element of respiration. In a chemical sense—and it is this alone which the preceding marks are intended to shew—caffeine or theine, asparagine and theobromine are, in virtue of their composition, better adapted to this purpose, than all other nitrogenised vegetable principles. The action of these substances, in ordinary circumstances, is not obvious, but it unquestionably exists. This part of our author's work is concluded by some ingenious observations on the theory of the action of the vegetable alkalies; the composition and origin of nervous matter; its relation to that of the vegetable alkalies, and the theory of the action of the latter. The extent to which we have carried our analysis, however, prevents us from dwelling longer on this part of the work.

For a somewhat similar reason, we must decline introducing any of the third part of this work, which treats of the abstruse laws of the phenomena of motion, and is entirely of a speculative character. We shall therefore proceed to the analysis of our author's treatises on the *Theory of Disease*, and the *Theory of Respiration*.

### Theory of Disease.

Every substance or matter, every chemical or mechanical agency, which changes or disturbs the restoration of the equilibrium between the manifestations of the causes of waste and supply, in such a way as to add its action to the causes of waste, is called by our author *a cause of disease*. *Disease* occurs when the sum of vital force, which tends to neutralise all causes of disturbance (in other words, when the resistance offered by the vital force), is weaker than the acting cause of disturbance. Death is that condition where all resistance on the part of the vital force entirely ceases. To the observer, the action of a cause of disease exhibits itself in the disturbance of the proportions between waste and supply which is proper to each period of life. In medicine, every abnormal condition of supply or waste, in all parts or in a single part of the body, is called disease. It is evident that one and the same cause of disease will produce in the organism very different effects, according to the period of life; and that a certain amount of disturbance, which produces disease in the adult state, may be without influence in childhood or in old age. A cause of disease may, when it is added to the cause of waste in old age, produce death (annihilate all resistance on the part of the vital force); while in the adult state it may produce only a disproportion between supply and waste; and in infancy only an equilibrium between supply and waste.

A cause of disease which strengthens the causes of supply, either directly or indirectly, by weakening the action of the causes of waste, destroys, in the child and in the adult, the relative normal state of health; while in old age it merely brings the waste and supply into equilibrium.

A child, lightly clothed, can bear cooling by a low external temperature without injury to health; the force available for mechanical purposes and the temperature of its body increase with the change of matter which follows the cooling; while a high temperature, which impedes the change of matter, is followed by disease.

On the other hand, in hospital and charitable institutions in which old persons spend the last years of life, when the temperature of the dormitory, in Winter, falls even a few degrees below the usual point, we see that by this slight degree of cooling the death of the oldest and weakest is occasioned.

A deficiency of resistance, in a living part, to the causes of waste, is a deficiency of resistance to the action of the oxygen. When this resistance diminishes in a living part, the change of matter increases in an equal degree. Since the phenomena of motion in the animal body are dependant on the change of matter, the increase of the change of matter is followed by an increase of all the motions. According to the conducting power of the nerves, the available force is carried away by the nerves of involuntary motion alone, or by all the nerves together. Hence, if in consequence of a diseased transformation of living tissues, a greater amount of force be generated than is required for the production of the normal motions, it is seen in an acceleration of all or some of the involuntary motions, as well as in a higher temperature of the diseased part. This condition is called *fever*. When a great excess of force is produced by change of matter, the force, since it can only be consumed by motion, extends itself to the apparatus of voluntary motion. This state is called a *febrile paroxysm*. From the acceleration of the circulation in fever, more arterial blood, and in consequence, more oxygen, is conveyed to the diseased part, as well as to all other parts; and, if the active force in the healthy parts continue uniform, the whole action of the excess of oxygen must be exerted on the diseased part alone.

Should there be formed in the diseased parts, in consequence of the change of matter, from the elements of the blood or of the tissue, new products, which the neighbouring parts cannot employ for their own vital functions;—should the surrounding parts, moreover, be unable to convey these products to other parts, where they may undergo transformation, then these new products will suffer, at the place of their formation, a process of decomposition analogous to fermentation or putrefaction. In some cases medicine removes these diseased conditions, by exciting in the vicinity of the diseased part, or in any convenient situation, an artificial diseased state (as by blisters, &c.); thus diminishing, by means of artificial disturbance, the resistance offered to the external causes of change in these parts by the vital force. The physician succeeds in putting an end to the original diseased condition, when the disturbance artificially exerted exceeds in amount the diseased state to be overcome. The resistance offered by the vital force to the action of oxygen in the diseased part being feebler than in the healthy state, by artificially diminishing the resistance in another part, the chemical action is diminished in the diseased part, being directed to another part, where the physician has succeeded in producing a still more feeble resistance to the action of oxygen, (the change of matter).

Health and the restoration of the diseased tissue follow when we are able so far to weaken the disturbing action of oxygen, that it becomes inferior to the resistance of the vital force. In other cases, where artificial external disturbance produces no effect, other indirect methods are adopted to exalt the resistance offered by the vital force. The physician diminishes by blood-letting the number of the carriers of oxygen (the globules), and by this means the conditions of change of matter: he excludes from the food whatever is convertible into blood; he gives non-azotised food which supports the respiratory process, as well as fruit and vegetables, which contain the alkalies necessary for the secretions.

If by these means he succeeds in causing the vital force in the diseased part to overcome the chemical action, and that without arresting the functions of the other organs, health is restored. To this plan of cure there is a powerful ally for the diseased organ, viz. the vital force of the healthy parts. When blood is abstracted, the external causes of change are diminished in them, and so their vital force obtains the predominance—the sum of all resisting powers, in the system, taken together, increases in proportion as the amount of oxygen acting on them in the blood is diminished.

The vital force, in all its manifestations, behaves itself, like all other natural forces; it is devoid of consciousness or volition, and is subject to the action of a blister. According to the author's theory, the body may be compared to a self-regulating steam-engine with respect to the production of heat and of force. With the lowering of the external temperature the respirations become deeper and more frequent; oxygen is supplied in greater quantity; the change of matter is increased, and more food must be supplied, if the temperature of the body is to remain unchanged.

From the theory of disease here proposed it follows that a diseased condition once established, in any part of the body, cannot be made to disappear by the chemical action of a remedy. A limit may be put by a remedy to an abnormal process of transformation: that process may be accelerated or retarded; but this alone does not restore the healthy condition. The art of the physician consists in the knowledge of the means which enable him to exercise an influence on the *duration* of the disease; and in the removal of all disturbing causes, the action of which strengthens or increases that of the actual cause of disease.

The same method of treatment may restore health in one individual, which may prove fatal to another. Thus, in certain inflammations affecting highly muscular subjects, the antiphlogistic treatment is esteemed; while in other cases bloodletting is prejudicial. The vivifying agency of the blood must ever continue to be the most important condition in the restoration of a disturbed equilibrium, which result always depends on a saving of time. It is obvious, moreover, that in all diseases where the formation of contagious matter and of exanthemata is accompanied by fever, two diseased conditions simultaneously exist, and two processes are simultaneously completed; and that the blood, as it were by re-action (fever), becomes a means of cure, as being the carrier of that substance without whose aid the diseased products cannot be rendered harmless, destroyed, or expelled from the body; a means of cure, by which, in short, neutralization or equilibrium is effected.

### Theory of Respiration.

During the passage of venous blood through the lungs, the globules change their colour, and with this change, oxygen is absorbed—for every volume of oxygen absorbed, an equal volume of carbonic acid is, in most cases, given out. The red globules contain a *compound of iron*, and no other constituent of the body contains iron. The change of colour in the globules of venous blood depends on the action of oxygen.

The globules of arterial blood retain their colour in the larger vessels, and lose it during their passage through the capillaries. The change of colour in the venous globules depends on the combination of some one of their elements with oxygen; which absorption is attended with the separation of carbonic acid gas. This gas is not separated from the serum; as the serum has not the property of giving off carbonic acid, when in contact with oxygen.

Arterial blood when drawn from the body has its florid colour soon changed to a dark red—this is effected by the action of carbonic acid on the globules, florid blood absorbing a number of gases, which do not dissolve in the fluid part of the blood, when separated from the globules. *It is evident, therefore, that the globules have the power of combining with gases.*

The globules darkened by carbonic acid again become florid in oxygen, with disengagement of carbonic acid. The globules of the blood contain a compound of iron. From the invariable presence of iron in red blood, we must conclude that it is indispensably necessary to animal life; and since we know that the globules take no share in the process of nutrition, they evidently play a part in the respiratory process. The compound of iron in the globules has the characters of an oxidized compound. The observations made on the very remarkable properties of the compounds of iron lead to the opinion that the globules of arterial blood contain a compound of iron saturated with oxygen, which, in the living blood, loses its oxygen during its passage through the capillaries. The same thing occurs when it is separated from the body and begins to be decomposed. The compound, rich in oxygen, passes, therefore, by the loss of oxygen into one less charged with that element. One of the products of oxidation formed in this process is carbonic acid.

The compound of iron in the venous blood possesses the property of combining with carbonic acid; and it is obvious, that the globules of the arterial blood, after losing a part of their oxygen, will, if they meet with carbonic acid, combine with that substance. When they reach the lungs, they will again take up the oxygen they have lost; for every volume of oxygen absorbed a corresponding volume of carbonic acid is separated; they will return to their former state; that is, they will again acquire the power of giving off oxygen, from the well-known facility with which compounds of the peroxide of iron give up oxygen.

According to these views, the globules of arterial blood, in their passage through the capillaries yield oxygen to certain constituents of the body. A small portion of this oxygen serves to produce the change of matter, and determines the separation of living parts, and their conversion into lifeless compounds, as well as the formation of the secretions and excretions. The greater part, however, of the oxygen is employed in converting into oxidized compounds the newly-formed substances, which no longer form part of the living tissues. Hence, in the animal organism, two processes of oxidation are going on; one in the lungs, the other in the capillaries. By the former, in spite of the degree of cooling, and of the increased evaporation, the constant temperature of the lungs is kept up; while the heat of the rest of the body is supplied by the latter. According to our author's views the iron contained in the blood is the grand means of conveying to the lungs the carbonic acid formed in the system.

Let us suppose that the globules lose their property of absorbing oxygen, and of afterwards giving up this oxygen and carrying off the resulting carbonic acid; such a hypothetical case of disease will soon become perceptible in the temperature and other vital phenomena of the body. The change of matter will be arrested, no lifeless compounds are separated, neither bile nor urine can be formed; and the temperature of the body must sink; this state of things soon arrests the process of nutrition, and sooner or later death must follow, unaccompanied, however, by febrile symptoms.

In concluding our remarks on this truly valuable accession to medical science, we beg to state that the physiological chemist, as well as the medical practitioner, will find in this work ideas and practical remarks of a decidedly novel character. It is a beautiful and, we believe, the first specimen of the great services which modern organic chemistry is capable of rendering to practical medicine—indeed, we have no hesitation in saying, that, from its appearance, physiology will date a new æra in her advancement.

In the Appendix to this work, from which, for obvious reasons, we have declined making any extracts, are contained a number of the most recent and accurate analyses, which constitute the evidence on which the author's conclusions are founded.

